# Supplementary material for: Antihistamines Potentiate Dexamethasone Anti-Inflammatory Effects. Impact on Glucocorticoid Receptor-Mediated Expression of Inflammation-Related Genes
Source: Cells. 2021 Nov 5;10(11):3026. doi: 10.3390/cells10113026 (PMC8617649; doi:10.3390/cells10113026)
Supplement: Supplementary file 1 [file cells-10-03026-s001.zip › cells-1432798-supplementary.pdf]

cells MDPI

Supplementary Material

Supplementary Table S1. Primers used for quantitative real-time PCR

Primer	Fw (5′-3′)	Rv (5′-3′)	Reference [1]
GILZ [h]	AATGCGGCCACGGATG	GGACTTCACGTTTCAGTGGACA	NM_000758
MKP1 [h]	CTGCCTTGATCAACGTCTCA	ACCCTTCCTCCAGCATTCTT	NM_004417.4
IL-8 [h]	CTGCGCCAACACAGAAATTA	ATTGCATCTGGCAACCCTAC	NM_000584
COX2 [h]	TTCAAATGAGATTGTGGGAAAATTGCT	AGATCATCTCTGCCTGAGTATCTT	NM_000963.1
GMCSF [h]	CATGTGAATGCCATCCAGGA	GGAGGTCAAACATTTCTGAGATGA	NM_000758
ACTB [h]	GGACTTCGAGCAAGAGATGG	AGCACTGTGTTGGCGTACAG	NM_001101.3
OPG [m]	GGAAACAGAGAAGCCACGCAA	GGTAGGAACAGCAAACCTGAAG	NM_008764.3
RANKL [m]	GGAGGATGAAACAAGCCTTTCA	TCCAACCATGAGCCTTCCATC	NM_011613.3
OC [m]	GCTCTGTCTCTCTGACCTCACA	GGTAGGAACAGCAAACCTGAAG	NM_007541.3
ACTB [m]	AGACTTCGAGCAGGAGATGG	AGCACTGTGTTGGCATAGAG	NM_007393.3

[h] human; [m] murine; [1] NCBI (National Center for Biotechnology Information) entry.

Supplementary Figure S1

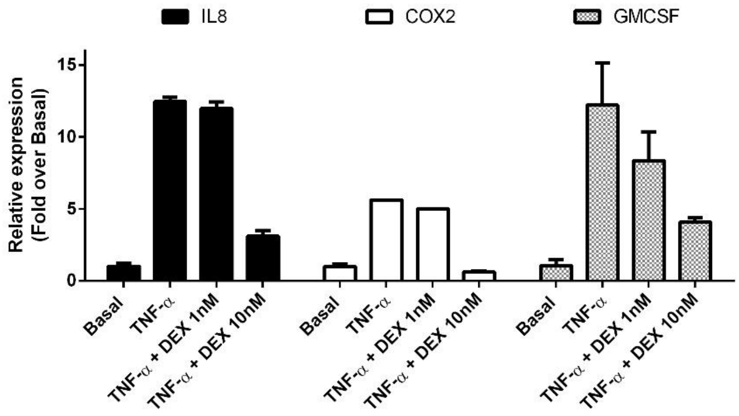

Figure S1. **Dexamethasone-induced transrepression of endogenous pro-inflammatory genes in A549 cells.** A549 cells were incubated with 2000 UI/ml TNFα for 4 h and then treated with dexamethasone (DEX) for 3 h, as indicated. IL-8, COX-2, and GMCSF mRNA levels were quantified by qPCR as described in the methods section of the main manuscript. Results are mean+/-SD of at least three independent experiments performed in triplicates.

Supplementary Figure S2

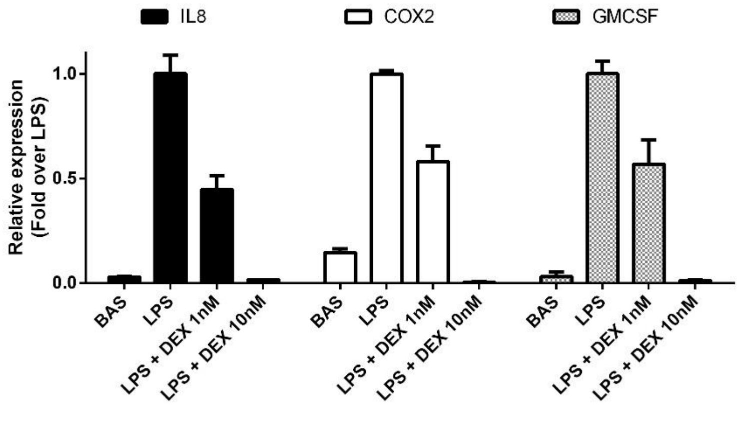

Figure S2. **Dexamethasone-induced transrepression of endogenous pro-inflammatory genes in U937 cells.** U937 cells were differentiated to macrophages with 100 nM PMA for 48 h and then stimulated with 1µg/µl LPS for 4 h. Then were exposed to dexamethasone (DEX) for 3 h, as indicated. IL-8, COX-2, and GMCSF mRNA levels were quantified by qPCR as described in the methods section of the main manuscript. Results are mean+/-SD of at least three independent experiments performed in triplicates.

Supplementary Figure S3

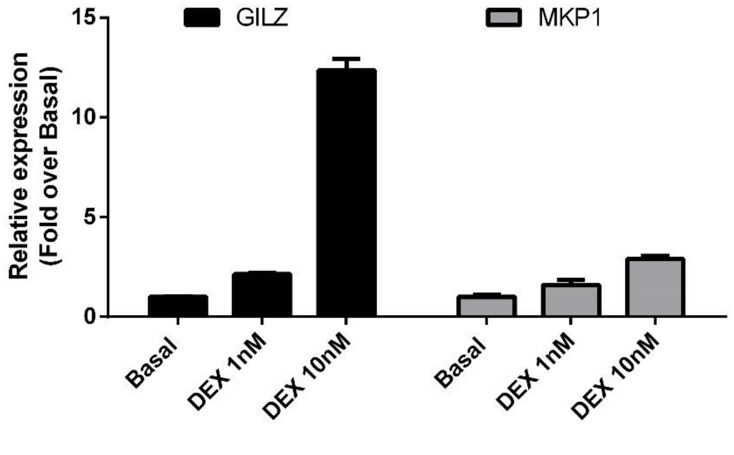

Figure S3. **Dexamethasone-induced expression of endogenous anti-inflammatory genes.** A549 cells were treated with dexamethasone (DEX) for 3 h, as indicated. GILZ and MKP1 mRNA levels were quantified by qPCR as described in the methods section of the main article. Results are mean+/-SD of at least three independent experiments performed in triplicates.

Supplementary Figure S4

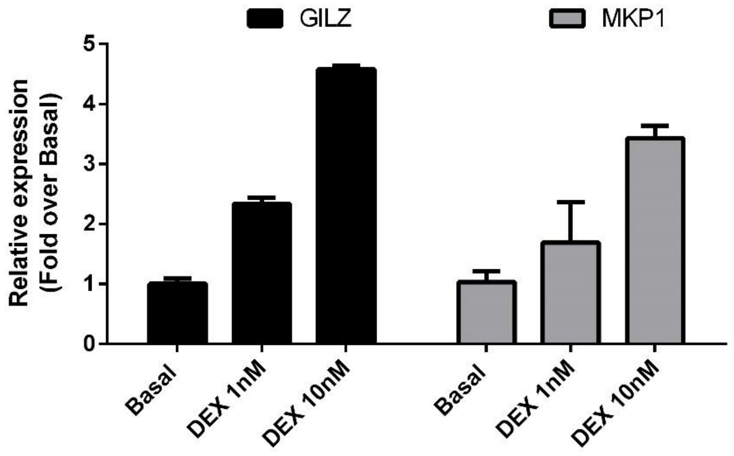

Figure S4. **Dexamethasone-induced expression of endogenous anti-inflammatory genes.** U937 cells were treated with dexamethasone (DEX) for 3 h, as indicated. GILZ and MKP1 mRNA levels were quantified by qPCR as described in the methods section of the main article. Results are mean+/-SD of at least three independent experiments performed in triplicates.
